# Supplementary material for: Comparative Transcriptomics Revealed Physalis floridana Rydb. Influences on the Immune System of the 28-Spotted Ladybird Beetle (Henosepilachna vigintioctopunctata)
Source: Plants (Basel). 2024 Sep 27;13(19):2711. doi: 10.3390/plants13192711 (PMC11478385; doi:10.3390/plants13192711)
Supplement: Supplementary file 1 [file plants-13-02711-s001.zip › plants-3155680-supplementary.pdf]

Table S1: Annotated result tables of 15 hub genes in Swiss-Prot, Pfam and NR

Gene_id	Swiss-Prot annotation	Pfam annotation	NR annotation
Unigene2681_All	Sarcotoxin-2A OS=Sarcophaga peregrina OX=7386 PE=1 SV=1	Attacin, C-terminal region	antibacterial peptide [Harmonia axyridis]
Unigene2682_All	NA	Attacin, C-terminal region	attacin 2 [Tenebrio molitor]
CL109.Contig4_All	Coleoptericin OS=Zophobas atratus OX=7074 PE=1 SV=1	Coleoptericin	coleoptericin [Anatolica polita]
Unigene9343_All	Attacin-A OS=Glossina morsitans morsitans OX=37546 PE=1 SV=1	Attacin, C-terminal region	uncharacterized protein BDFB_011474 [Asbolus verrucosus]
CL270.Contig2_All	NA	Attacin, C-terminal region	attacin 2 [Tenebrio molitor]
CL660.Contig1_All	NA	Attacin, C-terminal region	attacin 2 [Tenebrio molitor]
CL109.Contig6_All	Coleoptericin OS=Zophobas atratus OX=7074 PE=1 SV=1	Coleoptericin	coleoptericin [Anatolica polita]
Unigene4478_All	NA	Attacin, C-terminal region	antibacterial peptide [Harmonia axyridis]
CL270.Contig3_All	NA	Attacin, C-terminal region	attacin 2 [Tenebrio molitor]
CL270.Contig1_All	NA	Attacin, C-terminal region	attacin 2 [Tenebrio molitor]
CL109.Contig1_All	Coleoptericin OS=Zophobas atratus OX=7074 PE=1 SV=1	Coleoptericin	coleoptericin [Anatolica polita]
CL109.Contig3_All	Coleoptericin OS=Zophobas atratus OX=7074 PE=1 SV=1	Coleoptericin	coleoptericin [Anatolica polita]
Unigene5610_All	Defensin Lucifensin OS=Lucilia sericata OX=13632 PE=1 SV=2	NA	Defensin-like Protein [Tribolium castaneum]
CL109.Contig5_All	Coleoptericin OS=Zophobas atratus OX=7074 PE=1 SV=1	Coleoptericin	coleoptericin [Anatolica polita]
Unigene2683_All	Sarcotoxin-2A OS=Sarcophaga peregrina OX=7386 PE=1 SV=1	Attacin, C-terminal region	antibacterial peptide [Harmonia axyridis]

Table S2: Table of RT-qPCR primers

Gene ID	Forward-primer	Reverse- primer
HvRPL13	AGCATCCTTCGCTCGTTTAG	TTCGACAACCTGCCATTAGG
Unigene3049_All	CAGACGGGCCAGGACAAG	TGGATTCTGTGGCCGTGG
Unigene375_All	ACGATGCCTGTTGTGACGA	AACACCATACCGCTGCCG
Unigene9258_All	GTTGGTGTTGGAGGCTGGA	ACCACCAACGCACTCTGG
Unigene483_All	TGAGTGGAAGTTGGGATCACC	CCAGCAGTGCCTACCACC
Unigene209_All	GCAGGAGCAATGATGTCAGTG	TCGCCCTGCATTGGTGTT
Unigene4408_All	GTCGCAAGGACCGGTCAA	CCGGCTGATATGCACCCA
Unigene3992_All	CAGCGGTGGTGGGTTGAT	GCTGGCAGCAACGGAAAC
Unigene11687_All	CCAGAAGGTGGGGTCACTG	TGGCTCCATTGGGTCCAC
